# Supplementary material for: Catastrophic health expenditure: A comparative analysis of smoking and non-smoking households in China
Source: PLoS One. 2020 May 29;15(5):e0233749. doi: 10.1371/journal.pone.0233749 (PMC7259713; doi:10.1371/journal.pone.0233749)
Supplement: Attachment — Submitted filename: PLOSFeb2020.pdf [file pone.0233749.s001.pdf]

Review of "Catastrophic health expenditure: A comparative analysis of smoking and non-smoking households in China"

The authors use two waves of a household panel survey to assess the degree to which Chinese households face catastrophic health expenditure (CHE) related to smoking. First of all, they should provide an expanded Data Availability Statement. They indicate that the data are freely available and describe the CHARLS survey, but they do not provide a URL for this survey. The study analyzes the 2011 and 2013 waves of the survey; no indication is provided as to whether it could be expanded to include the 2015 or 2017 waves.

The authors use a measure of CHE developed in earlier studies (e.g., *Lancet* 2003, *WHO* 2005): essentially, that out-of-pocket health expenditures exceed 40% of "Capacity to Pay", defined as total household consumption net of food consumption. They define the mean gap (MG) as the average percentage of CHE over the survey sample, and the mean positive gap (MPG) as the ratio of mean gap to the fraction of the population facing CHE. Those figures are only used as descriptive measures in the empirical analysis, which is based on a panel logistic regression with random effects. Although there are repeated measures data on households, the only seeming contribution of the panel logit is the inclusion of a household-level random effect accounting for unobserved heterogeneity. The dichotomous nature of the outcome variable precludes consideration of the magnitude of CHE. It would also be interesting to see a tabulation of households never facing CHE, those experiencing it in one of the two years, and those experiencing it in both years, as the latter outcome would have a more devastating effect on the household's budget.

As the household-level gap is being computed for inclusion in the MG and MPG measures, it seems that its magnitude should be considered in the estimated model. The gap at the household-year level, $E_{it}(\frac{oop_{it}}{ctp_{it}} - 0.4)$, is a left-censored variable. One would surely expect the impact of CHE to differ depending on whether that ratio is 0.41, 0.51 or 0.61. It seems like a censored panel regression model would be more appropriate.

Finally, with regard to the policy recommendations: as elsewhere in the world, anti-smoking efforts such as publicity campaigns and higher tobacco taxes, as well as restrictions on smoking locales, would certainly be useful in reducing the incidence of smoking in China. Health benefits that assist current smokers to quit would be beneficial. The authors' point that the health damage from smoking often occurs with years' lag is well taken, but it is somewhat surprising that their only statistically significant findings relate to households with former smokers, and not those with current smokers. Higher taxes and other anti-smoking efforts will not have an effect on households with former smokers, but the financial burden of their past behavior is substantial. Policies to assist those households bear the burden of CHE should be a component of any anti-smoking intitative so that they are not impoverished by these expenditures.
