# Supplementary material for: Catastrophic health expenditure: A comparative analysis of smoking and non-smoking households in China
Source: PLoS One. 2020 May 29;15(5):e0233749. doi: 10.1371/journal.pone.0233749 (PMC7259713; doi:10.1371/journal.pone.0233749)
Supplement: Attachment — Submitted filename: Response to Reviewers.docx [file pone.0233749.s002.docx]

**Response to Reviewers**

**Response to comments by Lian Yang et al.**

About Funding Statement：

*We note that you have provided funding information that is not currently declared in your Funding Statement. However, funding information should not appear in the Acknowledgments section or other areas of your manuscript. We will only publish funding information present in the Funding Statement section of the online submission form.*

*Please remove any funding-related text from the manuscript and let us know how you would like to update your Funding Statement. Currently, your Funding Statement reads as follows:*

*"The funders had no role in study design, data collection and analysis, decision to publish, or preparation of the manuscript."*

**Response:**Funding Statement：This work was supported by National Natural Science Foundation of China, grant no. 71603032.The funders had no role in study design, data collection and analysis, decision to publish, or preparation of the manuscript.

*Reviewer #1: Overall, this manuscript provides an estimate of the incidence and severity of catastrophic financial impact on households from tobacco use in China. This is particularly interesting given the large total number of tobacco users in China, which contributes substantially to the global burden of disease. This survey is notable for its high response rate and broad geographic range regarding regions in China. I was surprised to see that current smoker households do not have a substantially higher incidence or proportion of catastrophic health expenditures than nonsmoker households, and some of my recommendations for the manuscript may provide better insight into that finding.*

**Response:**Thank you very much for your time and kindness. We hope that in this round of revision we could fully address all of your concerns.

*1) it would be helpful to have a copy editor review the manuscript to clarify minor issues with the English wording. It was also not easy to understand the difference between mean gap and mean positive gap-the formulas helped but it would be good to reframe these in the text so that it is clear the mean gap is the proportion of CHE for the group analyzed, and the mean positive gap is the proportion of CHE for those experiencing CHE within the group analyzed.*

**Response:** A good catch! Thank you for your helpful comments, which we are happy to follow. In response, the manuscript has been reviewed many times by copy editors to avoid minor problems in English wording. Regarding the intensity indicators of catastrophic health expenditure: the mean gap and mean positive gap, we have modified the formula, hoping to make it easier for you and other readers to understand and distinguish the two concepts. The new formula is as follows:

| $\text{MG}=\frac{1}{\text{N}_{1}}\sum_{\text{i}=1}^{\text{N}_{1}} \text{（}\frac{\text{OOP}}{\text{CTP}}\text{-0.4）}\times\text{CHE}_{\text{i}}$ | （1） |
| --- | --- |

where N1 is the total sample size (households incurring CHE or not)；and CHE_i_ means the probability of household i incurring CHE (0-No, 1-Yes).

| $\text{MPG}=\frac{1}{\text{N}_{2}}\sum_{\text{i}=1}^{\text{N}_{1}} \text{（}\frac{\text{OOP}}{\text{CTP}}\text{-0.4）}\times\text{CHE}_{\text{i}}$ | （2） |
| --- | --- |

where N2 is the sample size of household incurred CHE.

The corresponding part of the manuscript was amended to:

Methods

In previous studies, there have been two measurement criteria for CHE. When out-of-pocket healthcare payments (OOP) accounted for more than 10% of total household expenditures, CHE was considered to have occurred[23-25]. In addition, when OOP accounted for 40% or more of the household's Capacity to Pay (CTP), the health expenditure was also considered catastrophic[16,18,26-29]. This study adopts the second criterion. A household’s CTP is defined as the effective income after basic subsistence needs are satisfied. In many studies, effective income is considered to be the total household consumption expenditure, and basic subsistence needs refer to household food expenditures[16,30]. Therefore, the household’s CTP is equal to the result of total household consumption subtracted by household food expenditures[31-33].

The frequency and severity of CHE are often measured with the incidence and intensity of CHE. The incidence of CHE refers to the percentage of households with CHE in all households. The mean gap (MG) and mean positive gap (MPG) reflect the intensity of CHE[18-20]. The MG evaluates the severity of CHE in all sample households, while the MPG measures the severity of CHE in households with CHE. CHE_i_ is used to represent whether a household has CHE. The formula is as follows:

| $CHE_{i}=\left\{\begin{matrix} 1 if \frac{oop_{i}}{ctp_{i}}\geq0.4 \\ 0 if \frac{oop_{i}}{ctp_{i}}<0.4 \end{matrix}\right.$ | （1） |
| --- | --- |

$oop_{i}$represents the OOP of the “i-th” family;$ctp_{i}$represents the capacity of the “i-th” family to pay. The incidence and intensity of CHE are calculated as follows :^[34 ,35]^

| $H=\frac{1}{N_{1}}\sum_{i=1}^{N_{1}} CHE_{i}$ | （2） |
| --- | --- |
| $\text{MG}=\frac{1}{\text{N}_{1}}\sum_{\text{i}=1}^{\text{N}_{1}} \text{（}\frac{\text{OOP}}{\text{CTP}}\text{-0.4）}\times\text{CHE}_{\text{i}}$ | （3） |
| $\text{MPG}=\frac{1}{\text{N}_{2}}\sum_{\text{i}=1}^{\text{N}_{1}} \text{（}\frac{\text{OOP}}{\text{CTP}}\text{-0.4）}\times\text{CHE}_{\text{i}}$ | （4） |

N_1_ represents the total sample size (households incurring CHE or not), N_2_ represents the sample size of household incurred CHE, $\text{CHE}_{\text{i}}$ means the probability of household i incurring CHE (0-No, 1-Yes), and H represents the incidence of CHE.

*2) it could be clearer which regions are sampled. Instead of naming four regions not included in the sampling frame on page 12, it would be easier to state the 28 regions (first all listed on page 15)*

**Response:** A good comment, and we are happy to follow. We will show all 28 provinces / municipalities / autonomous regions, and the sampling regions will be clear at a glance. Accordingly, we have modified the data source. The new data source is as follows:

“**Data source**

Our research data are mainly derived from the China Health and Retirement Longitudinal Study (CHARLS). CHARLS is a national, large-scale follow-up project launched in 2011 and tracked once every two years. The survey used a multistage and Probabilities Proportional to Size(PPS) sampling strategy to collect data from 28 provinces/municipalities/autonomous regions in the country (provinces/municipalities/autonomous regions including Sichuan, Chongqing, Guizhou, Yunnan, Gansu, Guangxi, Inner Mongolia Autonomous Region, Qinghai, Shaanxi, Xinjiang Uygur Autonomous Region, Shanxi, Jilin, Heilongjiang, Anhui, Henan, Hubei, Hunan , Jiangxi, Beijing, Tianjin, Hebei, Liaoning, Fujian, Guangdong, Jiangsu, Shandong, Shanghai and Zhejiang were sampled). The survey subjects are households with members aged 45 and above. With a large sample size covering most parts of the country and different aspects, the CHARLS survey questionnaire is able to provide all the data needed to calculate CHE. Having retained individual and household data tracked in 2011 and 2013, we successfully retrieved balance panel data covering 8073 households with a follow-up rate of 78.74%. ”

*3) I am not totally clear if this is a pooled regression analysis rather than a panel regression analysis that uses the respondents that were in both waves of the survey. I am not sure what was the random effect? I would have thought it was time but then it appeared in the table.*

**Response:** A good catch!“random effect” is synonymous with zero correlation between the observed explanatory variables and the unobserved effect: Cov(x_it_, c_i_)=0, t=1,2,...,T. [Actually, a stronger conditional mean independence assumption, E(ci|x_i1_,...,x_iT_)=E(c_i_),will be needed to fully justify statistical inference.] In applied papers, when c_i_ is referred to as, say, an ”individual random effect” , then c_i_ is probably assumed to be uncorrelated with the x_it_.

Smoking is addictive, and smoking behaviors are difficult to change. Analyzing them with the select panel data fixed effect model will result in a loss of samples with unchanged smoking status, fitting model parameters that deviate from reality. For panel data, pooled effects models usually think that the estimation is biased.To ensure the robustness of the analysis results, this study used the random-effects model.

We changed the family size from the previous categorical variable (1-2 persons, 3-4 persons, ≥5 persons) to a continuous variable in the model and replaced “Having hospitalized members in household” with “Having seriously ill members in household. A family whose member's total annual hospitalization cost exceeds5,000 yuan per person is considered to have suffered from a serious illness (yes, no)”.

We tried fixed-effects model, pooled regression analysis, and random-effects model for analysis, and finally adopted the results of random-effects model (Table 3). **Table 4 in the manuscript was updated to the results of Table 3 here**.

Table 1 Fixed-Effects Logit Model Results of Influencing Factors of Chinese Household CHE

| CHE | OR | Std. Err. | z | P>z | [95% Conf.Interval] | |
| --- | --- | --- | --- | --- | --- | --- |
| Current smoking household vs. Non-smoking household | 1.123 | 0.238 | 0.55 | 0.584 | 0.7410929 | 1.702673 |
| Former smoking household vs. Non-smoking household | 1.573 | 0.320 | 2.22 | **0.026** | 1.055295 | 2.343469 |
| Urban area vs. Rural area | 1.000 | (omitted) |  |  |  |  |
| Having seriously ill members in household（Yes vs. No） | 6.941 | 0.977 | 13.77 | **<0.001** | 5.26828 | 9.145341 |
| Household size | 0.853 | 0.041 | -3.31 | **0.001** | 0.7759928 | 0.9370989 |
| Having medical insurance covered members in household（Yes vs. No） | 1.162 | 0.266 | 0.66 | 0.511 | 0.7426145 | 1.818526 |
| Poorer vs. Poorest | 0.939 | 0.110 | -0.54 | 0.591 | 0.745885 | 1.181637 |
| Middle vs. Poorest | 0.840 | 0.110 | -1.33 | 0.184 | 0.6489802 | 1.08656 |
| Richer vs. Poorest | 0.840 | 0.126 | -1.16 | 0.245 | 0.6251677 | 1.127517 |
| Richest vs. Poorest | 0.852 | 0.150 | -0.91 | 0.364 | 0.603719 | 1.203531 |
| Having elderly people aged 65 and above in household（Yes vs. No） | 0.968 | 0.191 | -0.16 | 0.871 | 0.6580758 | 1.425233 |
| Having chronic disease members in household（Yes vs. No） | 5.175 | 2.499 | 3.4 | **0.001** | 2.008455 | 13.33507 |
| Having disabled members in household（Yes vs. No） | 1.041 | 0.092 | 0.46 | 0.647 | 0.8756746 | 1.238326 |
| Having alcoholic members in household（Yes vs. No） | 0.793 | 0.099 | -1.87 | 0.062 | 0.6209446 | 1.011495 |
| 2013 vs. 2011 | 1.094 | 0.067 | 1.48 | 0.139 | 0.9710261 | 1.233301 |
| The Middle vs. The West | 1.000 | (omitted) |  |  |  |  |
| The East vs. The West | 1.000 | (omitted) |  |  |  |  |

Table 2 Pooled Logit Model Results of Influencing Factors of Chinese Household CHE

| CHE | Odds Ratio | Std. Err. | z | P>z | [95% Conf.Interval] | |
| --- | --- | --- | --- | --- | --- | --- |
| Current smoking household vs. Non-smoking household | 1.133 | 0.066 | 2.12 | **0.034** | 1.010 | 1.271 |
| Former smoking household vs. Non-smoking household | 1.386 | 0.102 | 4.41 | **<0.001** | 1.199 | 1.602 |
| Urban area vs. Rural area | 0.788 | 0.044 | -4.29 | **<0.001** | 0.707 | 0.879 |
| Having seriously ill members in household（Yes vs. No） | 8.790 | 0.585 | 32.66 | **<0.001** | 7.715 | 10.015 |
| Household size | 0.804 | 0.013 | -13.06 | **<0.001** | 0.778 | 0.831 |
| Having medical insurance covered members in household（Yes vs. No） | 1.239 | 0.170 | 1.56 | 0.120 | 0.946 | 1.622 |
| Poorer vs. Poorest | 0.923 | 0.063 | -1.17 | 0.240 | 0.807 | 1.055 |
| Middle vs. Poorest | 0.779 | 0.057 | -3.4 | **0.001** | 0.674 | 0.900 |
| Richer vs. Poorest | 0.569 | 0.047 | -6.86 | **<0.001** | 0.484 | 0.669 |
| Richest vs. Poorest | 0.489 | 0.044 | -7.87 | **<0.001** | 0.409 | 0.584 |
| Having elderly people aged 65 and above in household（Yes vs. No） | 1.598 | 0.082 | 9.09 | **<0.001** | 1.444 | 1.768 |
| Having chronic disease members in household（Yes vs. No） | 4.103 | 0.563 | 10.29 | **<0.001** | 3.136 | 5.369 |
| Having disabled members in household（Yes vs. No） | 1.259 | 0.070 | 4.16 | **<0.001** | 1.129 | 1.403 |
| Having alcoholic members in household（Yes vs. No） | 0.918 | 0.048 | -1.64 | 0.101 | 0.829 | 1.017 |
| 2013 vs. 2011 | 1.012 | 0.050 | 0.23 | 0.818 | 0.918 | 1.115 |
| The Middle vs. The West | 0.953 | 0.056 | -0.82 | 0.411 | 0.849 | 1.069 |
| The East vs. The West | 0.800 | 0.049 | -3.64 | **<0.001** | 0.710 | 0.902 |
| _cons | 0.061 | 0.012 | -13.97 | **<0.001** | 0.041 | 0.091 |

Table 3 Logit Random Effects Model Results of Influencing Factors of Chinese Household CHE

| Variable | OR | Std.Error | z-Statistic | Prob. | 95% C.I. | |
| --- | --- | --- | --- | --- | --- | --- |
|  |  |  |  |  | Lower | Upper |
| Current smoking household vs. Non-smoking household | 1.160 | 0.085 | 2.03 | **0.042** | 1.005 | 1.338 |
| Former smoking household vs. Non-smoking household | 1.509 | 0.138 | 4.51 | **<0.001** | 1.262 | 1.805 |
| Urban area vs. Rural area | 0.747 | 0.052 | -4.2 | **<0.001** | 0.652 | 0.856 |
| Having chronic disease members in household（Yes vs. No） | 5.046 | 0.801 | 10.2 | **<0.001** | 3.697 | 6.887 |
| Having seriously ill members in household（Yes vs. No） | 13.737 | 1.292 | 27.86 | **<0.001** | 11.425 | 16.517 |
| Having medical insurance covered members in household（Yes vs. No） | 1.256 | 0.202 | 1.42 | 0.156 | 0.917 | 1.722 |
| Having elderly people aged 65 and above in household（Yes vs. No） | 1.755 | 0.115 | 8.62 | **<0.001** | 1.544 | 1.995 |
| Having disabled members in household（Yes vs. No） | 1.292 | 0.084 | 3.92 | **<0.001** | 1.137 | 1.469 |
| Having alcoholic members in household（Yes vs. No） | 0.888 | 0.056 | -1.88 | 0.061 | 0.784 | 1.005 |
| Household size | 0.777 | 0.016 | -12.31 | **<0.001** | 0.746 | 0.809 |
| Poorer vs. Poorest | 0.909 | 0.074 | -1.16 | 0.245 | 0.775 | 1.067 |
| Middle vs. Poorest | 0.750 | 0.066 | -3.26 | **0.001** | 0.631 | 0.892 |
| Richer vs. Poorest | 0.536 | 0.052 | -6.41 | **<0.001** | 0.443 | 0.648 |
| Richest vs. Poorest | 0.456 | 0.049 | -7.31 | **<0.001** | 0.369 | 0.563 |
| The Middle vs. The West | 0.937 | 0.070 | -0.87 | 0.385 | 0.809 | 1.085 |
| The East vs. The West | 0.763 | 0.059 | -3.51 | **<0.001** | 0.656 | 0.887 |
| 2013 vs. 2011 | 1.018 | 0.056 | 0.32 | 0.752 | 0.913 | 1.134 |
| _CONS | 0.035 | 0.008 | -13.92 | **<0.001** | 0.022 | 0.056 |

*4) As the unit of analysis is a household, a change in a single individual could be counted the same way as 5+ individuals. It may be helpful to see a sensitivity analysis where the costs are adjusted for individuals in the family rather than just using it as a fixed effect. This may help explain the surprising findings that households with current smokers do not have higher costs compared to nonsmoking households.*

**Response:** Thank you for your advice! We have made some adjustments to the independent variables of the model., We have changed the family size from the previous categorical variable (1-2 persons, 3-4 persons, ≥5 persons) to a continuous variable as shown in Table 3. The results of the new model show that the status of current smoking households is also a risk factor affecting the occurrence of catastrophic health expenditure. The model results refer to the response to question 3.

*5) One of the advantages of panel data is to evaluate effects among individuals undergoing transitions. it would be helpful if the study took advantage of the panel data available. Instead of analyzing only 3 categories (non, current, former) there could be analyses of seven categories based on 2011 and 2013 status (non to non, current to current, former to former, non to current, non to former, current to former, former to current). Given the reported rate of missingness, it is unclear how many people are transitioning from one state (nonsmoking/former/current) to another (nonsmoking/former/current). Also understanding these transitions would help explain why nonsmoking and current smoking households both decline between 2011 and 2013 which seems unusual as nonsmoking households can only become smoking households or stay nonsmoking, and not feed into the former category (unless allowed to have someone smoke for a year and then quit before the 2013 survey. The transition states may also help clarify the surprising findings for current smokers, as those who were current smokers in 2011 may have different trajectories for those in 2013 that become former smokers vs those that stay current smokers. Is it that groups transitioning from current to former have the highest proportion and severity of CHE?*

**Response:** In response to your suggestion, we will classify the smoking status in 2011 and 2013.Our study allowed to have someone smoke for a year and then quit before the 2013 survey. Smoking status changed in 19.05% of households(nonsmoking to current smoking, nonsmoking to former smoking, current smoking to former smoking, and former smoking to current smoking ).The proportion and severity of CHE in the transition from current smoking to the former smoking group is not the highest.(see table 4 and table 5). We also tried Logit regression analysis using the smoking variable 7 classification. The variables used were new variables(see table 6). Consistent with the results of the three-class model (table 3),the results show that the status changes from current smoking to current smoking, former smoking to former smoking and from former smoking to current smoking are more prone to CHE than that from non to non. are. This indicates that our results are robust.

Table 4 Changes in smoking status from 2011 to 2013

| Smoking status | Number of households | % |
| --- | --- | --- |
| non to non | 2091 | 25.90 |
| non to current | 185 | 2.29 |
| non to former | 334 | 4.14 |
| current to current | 3767 | 46.66 |
| current to former | 645 | 7.99 |
| former to former | 677 | 8.39 |
| former to current | 374 | 4.63 |
| Total | 8073 | 100.00 |

Table 5 Incidence, MG and MPG in different smoking status from 2011 to 2013

| Smoking status | Incidence | MG | MPG |
| --- | --- | --- | --- |
| non to non | 13.68 | 4.28 | 31.31 |
| non to current | 12.43 | 2.85 | 22.92 |
| non to former | 20.66 | 4.46 | 21.57 |
| current to current | 14.73 | 4.33 | 29.38 |
| current to former | 17.98 | 8.57 | 47.63 |
| former to former | 19.50 | 4.70 | 24.13 |
| former to current | 20.05 | 9.22 | 45.97 |
| Total | 15.56 | 4.88 | 31.40 |

Table 6 Results of logit regression model for smoking 7 classification

| Variable | OR | Std. Err. | z | P>z | [95% Conf. | Interval] |
| --- | --- | --- | --- | --- | --- | --- |
| non to current vs.non to non | 1.087 | 0.2446 | 0.37 | 0.711 | 0.699 | 1.690 |
| non to former vs.non to non | 1.320 | 0.2072 | 1.77 | 0.077 | 0.971 | 1.796 |
| current to current vs.non to non | 1.182 | 0.0935 | 2.12 | **0.034** | 1.012 | 1.380 |
| current to former vs.non to non | 1.177 | 0.1467 | 1.30 | 0.192 | 0.921 | 1.502 |
| former to former vs.non to non | 1.403 | 0.1655 | 2.87 | **0.004** | 1.113 | 1.768 |
| former to current vs.non to non | 1.547 | 0.2265 | 2.98 | **0.003** | 1.161 | 2.061 |
| Urban area vs. Rural area | 0.747 | 0.0516 | -4.22 | **<0.001** | 0.652 | 0.855 |
| Having seriously ill members in household（Yes vs. No） | 13.604 | 1.2682 | 28.00 | **<0.001** | 11.332 | 16.331 |
| Household size | 0.780 | 0.0159 | -12.24 | **<0.001** | 0.749 | 0.811 |
| Having medical insurance covered members in household（Yes vs. No） | 1.250 | 0.2000 | 1.40 | 0.162 | 0.914 | 1.711 |
| Poorer vs. Poorest | 0.911 | 0.0740 | -1.15 | 0.252 | 0.777 | 1.068 |
| Middle vs. Poorest | 0.751 | 0.0657 | -3.27 | **0.001** | 0.632 | 0.891 |
| Richer vs. Poorest | 0.539 | 0.0521 | -6.39 | **<0.001** | 0.446 | 0.651 |
| Richest vs. Poorest | 0.460 | 0.0491 | -7.27 | **<0.001** | 0.373 | 0.567 |
| Having elderly people aged 65 and above in household（Yes vs. No） | 1.751 | 0.1139 | 8.61 | **<0.001** | 1.541 | 1.989 |
| Having chronic disease members in household（Yes vs. No） | 5.022 | 0.7923 | 10.23 | **<0.001** | 3.687 | 6.842 |
| Having disabled members in household（Yes vs. No） | 1.296 | 0.0842 | 4.00 | **<0.001** | 1.141 | 1.472 |
| Having alcoholic members in household（Yes vs. No） | 0.895 | 0.0562 | -1.77 | 0.076 | 0.791 | 1.012 |
| 2013 vs. 2011 | 1.034 | 0.0565 | 0.62 | 0.538 | 0.929 | 1.151 |
| The Middle vs. The West | 0.938 | 0.0699 | -0.86 | 0.392 | 0.811 | 1.086 |
| The East vs. The West | 0.765 | 0.0588 | -3.49 | **<0.001** | 0.658 | 0.889 |
| _CONS | 0.035 | 0.0083 | -13.97 | **<0.001** | 0.022 | 0.056 |

*6) I am unclear what is a meaningful difference for Table 3.*

**Response:**Thanks for your nice comment. Table 3 in the manuscript is only a descriptive presentation of the relationship between family smoking status and CHE. Only after using logit or tobit models to control the influence of other factors, can we truly reveal the effect of family smoking status on the probability and degree of CHE. After we adjusted two variables（family size was adjusted from the previous categorical variable to a continuous variable in the model; “Having hospitalized members in household” was replaced by “Having seriously ill members in household），the logit model and tobit model results show that current and former smoking households (OR=1.160, P=0.042; OR=1.509, P<0.001) were more prone to catastrophic health expenditures than nonsmoking households, and that the current smoking households (P=0.02) and the former smoking households (P<0.001) can significantly increase the positive gap. The differences in Table 3 are significant.

*Reviewer #2:Review of “Catastrophic health expenditure: A comparative analysis of smoking and non-smoking households in China”:*

*Ⅰ.The authors use two waves of a household panel survey to assess the degree to which Chinese households face catastrophic health expenditure (CHE) related to smoking. First of all, they should provide an expanded Data Availability Statement. They indicate that the data are freely available and describe the CHARLS survey, but they do not provide a URL for this survey. The study analyzes the 2011 and 2013 waves of the survey; no indication is provided as to whether it could be expanded to include the 2015 or 2017 waves.*

**Response:**Thank you very much for your time and kind words. We hope that in this round of revisions we have fully addressed all of your concerns. We have added the Data Availability Statement and the URL, Some Details are as follows:

**Data Availability Statement:**

The family level and personal level data of people aged 45 and above in our study are all from China Health and retirement longitudinal study (CHARLS). The data users of CHARLS are all over the world. Interested researchers may contact and apply for data via the following contact information: Email:charls_intcrvicw@pku.cdu.cn; Tel: 400-610-1866.

**URL:**<http://charls.pku.edu.cn/>.Interested researchers can request data by clicking the open the URL and follow instructions .

It should also be added that CHARLS has conducted three follow-up surveys in 2011, 2013 and 2015 respectively. In the 2015 survey, there were missing variables such as household size and household income. As the quality of the data was not very good, it was not included in this study.

*Ⅱ.The authors use a measure of CHE developed in earlier studies (e.g., Lancet 2003, WHO 2005): essentially, that out-of-pocket health expenditures exceed 40% of “Capacity to Pay”, defined as total household consumption net of food consumption. They define the mean gap (MG) as the average percentage of mean gap to the fraction of the population facing CHE. Those figures are only used as descriptive measures in the empirical analysis, which is based on a panel logistic regression with random effects.Although there are repeated measures data on households, the only seeming contribution of the panel logit is the inclusion of a household-level random effect accounting for unobserved heterogeneity. The dichotomous nature of the outcome variable precludes consideration of the magnitude of CHE.It would also be interesting to see a tabulation of households never facing CHE, those experiencing it in one of the two years, and those experiencing it in both years, as the latter outcome would have a more devastating effect on the household’s budget.*

**Response:**Thanks for your good comment. As for what you mentioned: "t would also be interested to see a tabulation of houses never facing Che, those experiencing it in one of the two years, and those experiencing it in both years, as the lattice outcome would have a more developing effect on the house's budget", we are also interested. Following your suggestion, we have divided families into three categories: those who have never had CHE, those who have had CHE in one year, and those who have had CHE in two years. We also compared and analyzed the indicators of economic status of families. The results are consistent with your conjecture: household consumption expenditures, household food expenditures, and household payment capabilities decrease as the number of catastrophic health expenditures increases; household medical cash expenditures are just the opposite case: the more catastrophic health expenditures occur, the higher the cost (table 7) is.We have tried to use ordered logistic regression to analyze the influencing factors. The data results did not converge and the results could not be obtained. Panel Poisson regression was used for analysis (table 8). It should be noted that Poisson regression is generally used for rare events, and we do not consider it suitable for this study.

The detailed results are shown in the table below：

Table 7 The frequency of household catastrophic health expenditure and household economic situation(USD)

| Year | Frequency | Household type | Household consumption expenditure | | Household food expenditure | | Household payment capacity | | Household out of pocket health payment | |
| --- | --- | --- | --- | --- | --- | --- | --- | --- | --- | --- |
|  |  |  | Mean | S.D | Mean | S.D | Mean | S.D | Mean | S.D |
| 2011 | 0 | Non-smoking household | 3744 | 5230 | 1545 | 1858 | 2198 | 4608 | 112 | 273 |
|  |  | Current smoking household | 4006 | 5873 | 1870 | 2841 | 2135 | 4758 | 116 | 282 |
|  |  | Former smoking household | 4100 | 5733 | 1583 | 1554 | 2517 | 5327 | 160 | 297 |
|  |  | Total | 3931 | 5653 | 1729 | 2429 | 2201 | 4782 | 120 | 281 |
|  | 1 | Non-smoking household | 3131 | 4622 | 1130 | 1425 | 2001 | 4277 | 682 | 1989 |
|  |  | Current smoking household | 3186 | 3730 | 1409 | 1577 | 1777 | 3233 | 470 | 749 |
|  |  | Former smoking household | 3203 | 3543 | 1269 | 1670 | 1934 | 2819 | 693 | 1219 |
|  |  | Total | 3172 | 3992 | 1303 | 1552 | 1869 | 3526 | 569 | 1320 |
|  | 2 | Non-smoking household | 2142 | 2114 | 942 | 1208 | 1200 | 1536 | 802 | 1361 |
|  |  | Current smoking household | 2625 | 2590 | 1085 | 1184 | 1540 | 2042 | 991 | 1458 |
|  |  | Former smoking household | 3429 | 4056 | 910 | 969 | 2519 | 3802 | 1833 | 3385 |
|  |  | Total | 2630 | 2842 | 1005 | 1152 | 1625 | 2410 | 1098 | 2000 |
| 2013 | 0 | Non-smoking household | 4357 | 6013 | 1742 | 2255 | 2615 | 5051 | 162 | 444 |
|  |  | Current smoking household | 4792 | 7668 | 2210 | 4037 | 2582 | 5943 | 146 | 382 |
|  |  | Former smoking household | 5440 | 10439 | 2013 | 3872 | 3427 | 9280 | 219 | 602 |
|  |  | Total | 4775 | 7796 | 2040 | 3583 | 2734 | 6413 | 163 | 445 |
|  | 1 | Non-smoking household | 3499 | 6711 | 1198 | 1415 | 2301 | 6249 | 807 | 1634 |
|  |  | Current smoking household | 3870 | 6668 | 1584 | 2306 | 2286 | 5979 | 908 | 1733 |
|  |  | Former smoking household | 4475 | 5538 | 1582 | 1901 | 2893 | 4608 | 1433 | 2625 |
|  |  | Total | 3895 | 6471 | 1481 | 2029 | 2413 | 5804 | 988 | 1937 |
|  | 2 | Non-smoking household | 2435 | 2573 | 840 | 1506 | 1594 | 1817 | 1153 | 1596 |
|  |  | Current smoking household | 3120 | 3766 | 1375 | 2902 | 1745 | 1919 | 1139 | 1334 |
|  |  | Former smoking household | 3646 | 4056 | 1173 | 1633 | 2473 | 3060 | 1699 | 2465 |
|  |  | Total | 3051 | 3572 | 1186 | 2350 | 1865 | 2216 | 1267 | 1722 |

Table 8 Panel Poisson regression on the number of catastrophic health expenditures

| Variable | IRR | Std. Err. | z | P>z | [95% Conf. | Interval] |
| --- | --- | --- | --- | --- | --- | --- |
| Current smoking household vs. Non-smoking household | 1.072 | 0.057 | 1.31 | 0.19 | 0.966 | 1.190 |
| Former smoking household vs. Non-smoking household | 1.172 | 0.074 | 2.53 | **0.011** | 1.036 | 1.326 |
| Urban area vs. Rural area | 0.849 | 0.045 | -3.1 | **0.002** | 0.766 | 0.942 |
| Having seriously ill members in household（Yes vs. No） | 1.716 | 0.087 | 10.62 | **<0.001** | 1.553 | 1.895 |
| Household size | 0.876 | 0.012 | -9.45 | **<0.001** | 0.852 | 0.900 |
| Having medical insurance covered members in household（Yes vs. No） | 1.078 | 0.112 | 0.72 | 0.472 | 0.879 | 1.321 |
| Poorer vs. Poorest | 0.974 | 0.050 | -0.51 | 0.611 | 0.880 | 1.078 |
| Middle vs. Poorest | 0.913 | 0.052 | -1.61 | 0.107 | 0.817 | 1.020 |
| Richer vs. Poorest | 0.793 | 0.050 | -3.67 | **<0.001** | 0.700 | 0.897 |
| Richest vs. Poorest | 0.702 | 0.050 | -4.99 | **<0.001** | 0.611 | 0.806 |
| Having elderly people aged 65 and above in household（Yes vs. No） | 1.500 | 0.072 | 8.45 | **<0.001** | 1.365 | 1.648 |
| Having chronic disease members in household（Yes vs. No） | 2.719 | 0.275 | 9.87 | **<0.001** | 2.229 | 3.316 |
| Having disabled members in household（Yes vs. No） | 1.110 | 0.045 | 2.59 | **0.009** | 1.026 | 1.202 |
| Having alcoholic members in household（Yes vs. No） | 0.957 | 0.042 | -1.02 | 0.308 | 0.879 | 1.042 |
| 2013 vs. 2011 | 0.906 | 0.028 | -3.19 | **0.001** | 0.853 | 0.963 |
| The Middle vs. The West | 0.937 | 0.055 | -1.11 | 0.268 | 0.835 | 1.051 |
| The East vs. The West | 0.822 | 0.049 | -3.28 | **0.001** | 0.731 | 0.924 |
| _CONS | 0.156 | 0.024 | -12.04 | **<0.001** | 0.115 | 0.211 |

*Ⅲ.As the household-level gap is being computed for inclusion in the MG and MPG measures, it seems that its magnitude should be considered in the estimated model.The gap at the household-year level, Eit(oopit/ctpit-0.4), is a left-censored variable. One would surely expect the impact of CHE to differ depending on whether that ratio is 0.41, 0.51 or 0.61. It seems like a censored panel regression model would be more appropriate.*

**Response:**A good suggestion! In response, We have added this section to the manuscript. **The new content in this part is presented in Table 4 in the manuscript.**We used the Panel Tobit Regression with random effect to analyze the influencing factors of catastrophic health expenditure positive gap.

Table 9 Panel Tobit regression analysis of the positive gap of catastrophic health expenditure in China

| Variable | Coefficient | Std.Error | z-Statistic | Prob. | 95% C.I. | |
| --- | --- | --- | --- | --- | --- | --- |
|  |  |  |  |  | Lower | Upper |
| Current smoking household vs. Non-smoking household | 0.018 | 0.008 | 2.33 | **0.020** | 0.003 | 0.033 |
| Former smoking household vs. Non-smoking household | 0.036 | 0.010 | 3.49 | **<0.001** | 0.016 | 0.056 |
| Urban area vs. Rural area | -0.024 | 0.007 | -3.39 | **0.001** | -0.038 | -0.010 |
| Having chronic disease members in household（Yes vs. No） | 0.083 | 0.011 | 7.74 | **<0.001** | 0.062 | 0.104 |
| Having seriously ill members in household（Yes vs. No） | 0.317 | 0.012 | 26.88 | **<0.001** | 0.294 | 0.340 |
| Having medical insurance covered members in household（Yes vs. No） | 0.020 | 0.017 | 1.18 | 0.237 | -0.013 | 0.053 |
| Having elderly people aged 65 and above in household（Yes vs. No） | 0.042 | 0.007 | 5.75 | **<0.001** | 0.028 | 0.056 |
| Having disabled members in household（Yes vs. No） | 0.029 | 0.008 | 3.69 | **<0.001** | 0.013 | 0.044 |
| Having alcoholic members in household（Yes vs. No） | -0.016 | 0.007 | -2.42 | **0.015** | -0.030 | -0.003 |
| Household size | -0.011 | 0.002 | -5.67 | **<0.001** | -0.015 | -0.007 |
| Poorer vs. Poorest | 0.005 | 0.010 | 0.54 | 0.587 | -0.014 | 0.025 |
| Middle vs. Poorest | -0.005 | 0.010 | -0.52 | 0.603 | -0.025 | 0.015 |
| Richer vs. Poorest | -0.018 | 0.011 | -1.74 | 0.082 | -0.039 | 0.002 |
| Richest vs. Poorest | -0.039 | 0.011 | -3.53 | **<0.001** | -0.060 | -0.017 |
| The Middle vs. The West | -0.009 | 0.008 | -1.07 | 0.286 | -0.024 | 0.007 |
| The East vs. The West | -0.024 | 0.008 | -2.94 | **0.003** | -0.040 | -0.008 |
| 2013 vs. 2011 | 0.009 | 0.006 | 1.39 | 0.164 | -0.004 | 0.021 |
| _CONS | -0.316 | 0.021 | -14.79 | **<0.001** | -0.358 | -0.274 |

*Ⅳ.Finally, with regard to the policy recommendations: as elsewhere in the world, anti-smoking efforts such as publicity campaigns and higher tobacco taxes, as well as restrictions on smoking locales, would certainly be useful in reducing the incidence of smoking in China. Health benefits that assist current smokers to quit would be beneficial.The authors’ point that the health damage from smoking often occurs with years’ lag is well taken, but it is somewhat surprising that their only statistically significant findings relate to households with former smokers, and not those with current smokers. Higher taxes and other anti-smoking efforts will not have an effect on households with former smokers, but the financial burden of their past behavior is substantial. Policies to assist those households bear the burden of CHE should be a component of any anti-smoking intitative so that they are not impoverished by these expenditures.*

**Response:***According to the revised model results, both Tobit and Logit model results show that current smoking households are also more prone to catastrophic health expenditures. Tobacco control measures are also necessary. Although former smoking households are more serious, , the harm of corresponding former smoking households will be reduced accordingly if the current smoking population is controlled.*
